# Management and Follow-Up of Complicated Crown Fractures with Intrusive Luxation of Maxillary Incisors in an 8-Year-Old Boy

**DOI:** 10.1155/2021/5540860

**Published:** 2021-05-23

**Authors:** Niusha Abazarian, Shabnam Milani, Moahammad Hassan Hamrah, Marzieh Salehi Shahrabi

**Affiliations:** ^1^Department of Pediatric Dentistry, Dental School, AJA University of Medical Sciences, Tehran, Iran; ^2^Department of Pediatric Dentistry, Tehran University of Medical Sciences, Tehran, Iran; ^3^Department of Pediatric Dentistry, School of Dentistry, Tehran University of Medical Sciences Tehran, Iran

## Abstract

Intrusive luxation is a severe form of dental injury which causes damage to the pulp and supporting structures of a tooth because of its dislocation into the alveolar process. This paper shows the case of the reeruption of maxillary incisors accompanied by complicated crown fractures after 3 months. An 8-year-old boy patient was referred to the Department of Pedodontic Dentistry of Tehran University of Medical Science, Tehran, Iran, 18 hours after a fall at school. Clinical and radiographic examinations revealed intrusive luxation of both incisors with complicated crown fractures. Cervical pulpotomy is the treatment of choice for traumatized immature intruded teeth with pulp exposure. Two months later, the right central incisor teeth reerupted to a normal position and the final aesthetic restorations were done. The left central incisor was spontaneously repositioned with external root resorption, and the team decided to use interim medication (calcium hydroxide) in the root canal for stopping the process of resorption, and by the 9-month follow-up, the process of resorption had been stopped. An MTA plug was placed into the canal, and the final esthetic restorations were done.

## 1. Introduction

The most frequent traumatic dental injuries involving permanent teeth are complicated and uncomplicated crown fractures, and the most affected teeth are maxillary central incisors (93.3%) especially in children [1]. The most severe form of traumatic dental injury is intrusive luxation that accounts for 15–61% of traumas in permanent teeth and is defined as apical displacement of the tooth in its socket. The etiological factors include falling, bicycle injury, sports accidents, and fights [2, 3].

Intrusive luxation of permanent teeth is a rare dental injury when compared with other types of luxation injuries. It comprises of 3% of all traumatic injuries in the permanent teeth [1]. The displacement results in the damage to the alveolar bone, the periodontal ligament, the cementum, and the pulp. Healing subsequent to trauma is complex. Complications include pulp necrosis, inflammatory root resorption, dentoalveolar ankylosis, loss of marginal bone support, calcification of pulp tissue, paralysis or disturbance of root development, and gingival retraction [1, 4]. Intrusive luxations are associated with a high risk of complications during healing, including pulpal necrosis and calcification, external inflammatory resorption, replacement resorption, gingival retraction, and marginal bone loss [4]. The incidence of pulp necrosis for intruded teeth with open apex is significantly lower compared to closed apex, and it occurs between 63% and 68% in open apex and 100% for teeth with closed apex [1]. Depending on the severity of intrusion, the frequency of replacement resorption in intruded incisors ranges from 5% to 31% [2, 3] and appears to be more in mature than in immature teeth [5]. Although 97% of all inflammatory resorption are arrested after long-term calcium hydroxide therapy, there is no effective treatment for replacement resorption [1].

There are numerous ways to manage complicated crown fractures, such as direct pulp capping, partial pulpotomy, cervical pulpotomy, or pulpectomy. Furthermore, the treatment option for intruded teeth up to 7 mm for open apex is spontaneous repositioning [5].

Cervical pulpotomy is the treatment of choice for traumatized immature intruded teeth with pulp exposure. It allows the development of the roots to continue, with apical closing and strengthening of the root structure [6].

The present case report describes the management and follow-ups of traumatized immature intruded teeth with complicated crown fractures.

## 2. Case Presentation

**Figure 1:**
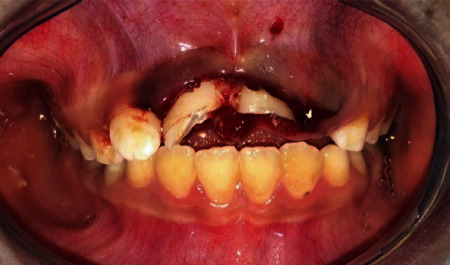
Intraoral examinations.

An 8-year-old boy patient was referred to the Department of Pedodontic Dentistry of Tehran University of Medical Science, Tehran, Iran, with the chief complaint being trauma of the central incisors following a fall at school 18 hours ago. The general medical, dental, and traumatic incident histories were recorded. There was no systemic disease history. Extraoral examination revealed abrasion on the skin of the chin and inflammation and bleeding of the labial gingival of the central incisors (Table 1). Intraoral examination revealed complicated crown fracture of the central incisors with no mobility and percussive metallic sound, indicating intrusion [1] (Figure 1).

**Figure 2:**
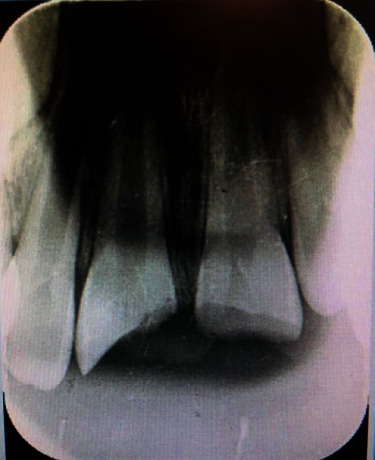
Radiographic examinations.

Periapical radiographic examination showed an intact periodontal ligament space, incomplete root formation of both central incisors, and no root fracture (Figure 2).

The periapical radiographies were taken using Xgenus (de Gotzen S.r.l device, distributed by Satelec-Acteon Group, Italy).

**Figure 3:**
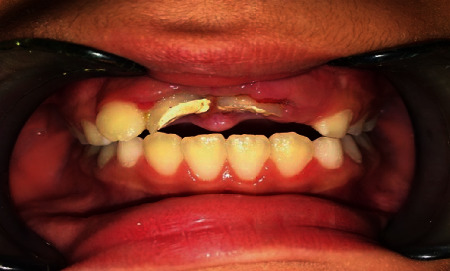
Cervical pulpotomy white MTA for both incisors.

Cervical pulpotomy using white mineral trioxide aggregate (BioMTA, Seoul, Republic of Korea) was done for both incisors after under local anesthesia and rubber dam isolation; all coronal pulp tissues were gently removed by using a high-speed sterile round diamond bur (Dentsply Maillefer, Tulsa, OK, USA) under water cooling. Hemorrhage was controlled with sterile cotton pellets and sterile saline solution to avoid clot formation. When pulpal bleeding stopped within 3 min, MTA powder was mixed with distilled water according to the recommended consistency and placed without any pressure to cover the exposed pulps. A moist cotton pellet was placed on the MTA, and the cavity was sealed temporarily with RMGI (Fuji IX, GC Corporation, Tokyo, Japan) (Table 2 and Figure 3).

**Figure 4:**
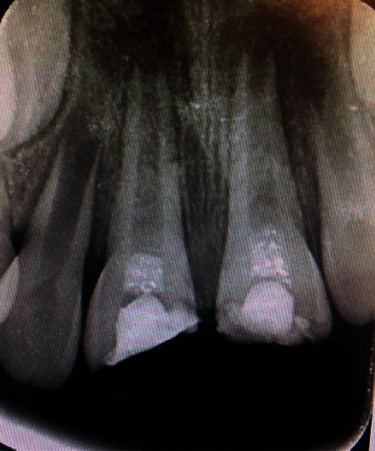
Follow-up after 4 weeks.

Follow-up after 4 weeks showed left central incisor percussion with spontaneous eruption, and the right central incisor percussion was metallic sound with no signs and symptoms. Radiographic evaluations have demonstrated a PDL widening in the middle third of the root in both central incisors (follow-up after 1 month) (Figure 4).

**Figure 5:**
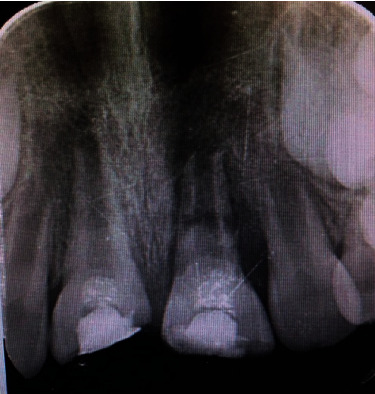
Follow-up after 8 weeks.

After 2 months of follow-up, the left center incisor was spontaneously repositioned with external root resorption and had a sensitive percussion. And the right central incisor with no signs and symptoms showed normal percussion and mobility with a normal radiography, and so, it was decided to do a composite buildup (Figure 5).

**Figure 6:**
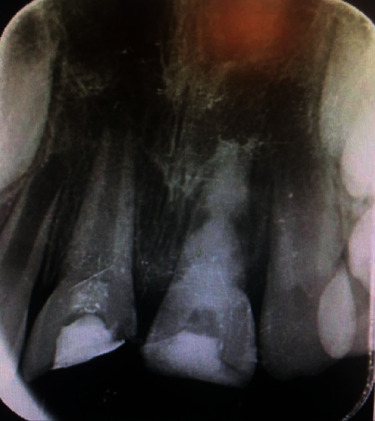
Calcium hydroxide was applied as intracanal medicament to stop the external resorption.

Treatment used to stop the external resorption of the left central incisor was irrigation with sodium hypochlorite (Beyond Technology Corp., Beijing, China) and saline. The CaOH (Prevest Denpro, Golchai, Iran) was positioned within the canal and dressed with RMGI (Fuji II LC, GC, Tokyo, Japan) (Figure 6).

**Figure 7:**
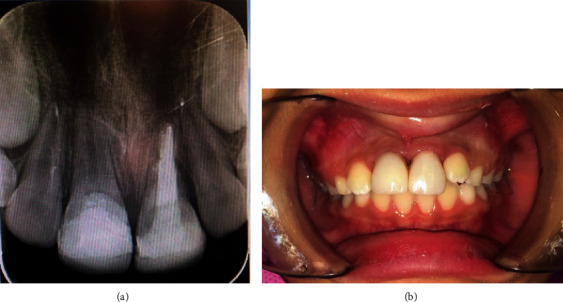
(a) MTA apical plug of 4-5 mm thickness and final obturation. (b) Final restoration of the tooth.

After 3 months, CaOH (Prevest Denpro, Golchai, Iran) was replaced. At the 9-month follow-up, the resorption process had stopped, and an MTA (BioMTA, Seoul, Republic of Korea) plug was placed in the canal and dressed with RMGI (Fuji II LC, GC, Tokyo, Japan). After one week, obturation, done with gutta-percha (reoko, Langenau, Germany) and composite (Filtek Z350 XT, 3 M ESPE, St. Paul, MN, USA) buildup, was performed (Figures 7(a) and 7(b)).

**Figure 8:**
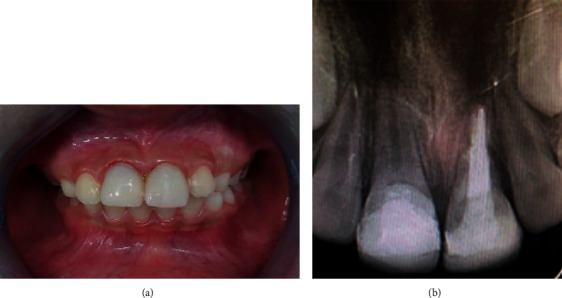
(a) Follow-up after 22 months. (b) Radiography after 22-month follow-up.

At the 22-month recall, the teeth were asymptomatic and showed no signs of resorption, clinically and radiographically (Figures 8(a) and 8(b)).

The patient has been followed for the last 22 months showing the success of the treatment (Table 3).

## 3. Discussion

Complicated crown fractures are defined as fractures involving enamel and dentin with pulp exposure. These injuries produce changes in the exposed pulp tissues, and a biological and functional restoration represents an important clinical challenge [1].

Dental traumas may include numerous injuries, including intrusion (33.5%), an associated crown fracture intrusion (60.5%), or a combination of intrusion and coronal or root fractures (6%) [2]. In most cases, it affects only one tooth (46.3%), followed by two teeth (32.4%) and three or more teeth (21.3%) [7]. Most of the intruded teeth are displaced from 1 to 8 mm into the alveolar bone by a traumatic force [8].

Intrusive luxation is a type of severe trauma that results in injury to the tooth structure, cells and fibers of periodontal ligament, pulp tissue, and alveolar bone [9].

Intrusive luxation is associated with a high risk of complications during healing and considered as one of the most difficult types of injury to treat as there are differing opinions on what constitutes as treatment. It was previously believed that the stage of development of the root was the determining factor for prognosis of intruded teeth [10]. Current dental literature suggests different treatment approaches for the management of intrusive luxation injuries including passive repositioning, allowing the tooth to reerupt, and active repositioning, either surgically or by use of orthodontic appliances [2]. Miniscrews are effective orthodontic devices for various orthodontic movements of the intruded teeth; it is recommended for dental extrusion without involving other teeth, implant side effects, or gingival margin cosmetic disability [11]. In case of intrusive luxation, the miniscrew-assisted orthodontic repositioning has been proposed [11]. In fact, orthodontic miniscrews demonstrated excellent mechanical properties and also with small diameters so they can be used also in hard to reach areas [12].

Root resorption after an intrusion is a commonly occurring scar complication. Inflammatory root resorption and replacement root resorption are classified as invasive or progressive root resorption [11, 12]. External root resorption has been reported and cited to be between 28% and 66% [1, 13, 14]. Andreasen et al. reported a total incidence of 86% of external resorption (38% inflammatory, 24% surface, and 24% replacement resorption). They also found a higher incidence of resorption in teeth with closed apices (70%) than in those with open apices (58%) [1].

It has been shown that calcium hydroxide stops the inflammatory resorption with a high degree of success [15]. Calcium hydroxide paste should be maintained in the root canal for 1 to 6 months before obturation with gutta-percha points. In this case, we applied calcium hydroxide (Prevest Denpro, Golchai, Iran) paste when the external root resorption has been started. After 9 months of follow-up, the resorption process had been stopped. In support of this, dressing to allow the healing should be kept for 6–9 months [16].

The long-term use of calcium hydroxide has some drawbacks. The treatment requires multiple appointments and takes anywhere from 3 to 18 months [17, 18]. It demands high cooperation and motivation from the patient. In addition, the long-term presence of calcium hydroxide in root canal space can increase the brittleness of the root dentin and the risk of future cervical root fractures especially in open apex teeth [11]. In spite of these disadvantages, it is still the preferred treatment protocol due to its high success rate [1, 11].

This case report shows that within the limitations of this study is a successful outcome. However, there were weaknesses on follow-up of the patient due to the COVID-19 pandemic lockdown and delay in attending patient to hospital after injury.

## 4. Conclusion

The findings in this case report suggest that calcium hydroxide stops the inflammatory resorption with a high degree of success.

## Figures and Tables

**Table 1:** Patient characteristics.

Age	Sex	Chief complaint	Clinical examination (extraoral examination)	Clinical examination (intraoral examination)	Radiographic examination
8 years old	Male	Trauma of the central incisors following a fall at school 18 hours ago	Abrasion on the skin of the chin inflammation and bleeding of the labial gingival of the central incisors	Complicated crown fracture of the central incisors with no mobility and percussive metallic sound	An intact periodontal ligament space, incomplete root formation, no root fracture

**Table 2:** Clinical passages.

Phase number	Treatment steps
1	Under local anesthesia and rubber dam isolation, all coronal pulp tissues were gently removed by using a high-speed sterile round diamond bur (Dentsply Maillefer, Tulsa, OK, USA) under water cooling.
2	Hemorrhage was controlled with sterile cotton pellets and sterile saline solution to avoid clot formation.
3	When pulpal bleeding stopped within 3 min, MTA powder was mixed with distilled water according to the recommended consistency and placed without any pressure to cover the exposed pulps.
4	A moist cotton pellet was placed on the MTA, and the cavity was sealed temporarily with RMGI (Fuji IX, GC Corporation, Tokyo, Japan).

**Table 3:** Clinical procedures.

Phase number	Treatment steps
First session	Cervical pulpotomy white MTA for both incisors
Follow-up after 4 weeks	(i) Left central incisor percussion with spontaneous eruption (ii) Right central incisor percussion was metallic sound with no signs and symptoms (iii) Radiographic evaluations have demonstrated a PDL widening in the middle third of the root in both central incisors
Follow-up after 8 weeks	(i) The left center incisor was spontaneously repositioned with external root resorption and had a sensitive percussion, and calcium hydroxide was applied as intracanal medicament (ii) The right central incisor with no signs and symptoms showed normal percussion and mobility with a normal radiography
Follow-up after 36 weeks	The resorption process had stopped, and an MTA plug was placed in the canal and dressed with RMGI.
Follow-up after 37 weeks	Obturation, done with gutta-percha and composite buildup, was performed
Follow-up after 22 months	The teeth were asymptomatic and showed no signs of resorption, clinically and radiographically.
